# MicroRNA-150 Is a Potential Biomarker of HIV/AIDS Disease Progression and Therapy

**DOI:** 10.1371/journal.pone.0095920

**Published:** 2014-05-14

**Authors:** Saif Ullah Munshi, Harekrushna Panda, Prasida Holla, Bharat Bhushan Rewari, Shahid Jameel

**Affiliations:** 1 Virology Group, International Centre for Genetic Engineering and Biotechnology, New Delhi, India; 2 ICGEB-Emory Vaccine Centre, International Centre for Genetic Engineering and Biotechnology, New Delhi, India; 3 ART Department, National AIDS Control Organization and Dr. Ram Manohar Lohia Hospital, New Delhi, India; University of Pittsburgh, United States of America

## Abstract

**Background:**

The surrogate markers of HIV/AIDS progression include CD4 T cell count and plasma viral load. But, their reliability has been questioned in patients on anti-retroviral therapy (ART). Five microRNAs (miRNAs) - miR-16, miR-146b-5p, miR-150, miR-191 and miR-223 in peripheral blood mononuclear cells (PBMCs) were earlier found to assign HIV/AIDS patients into groups with varying CD4 T cell counts and viral loads. In this pilot study, we profiled the expression of these five miRNAs in PBMCs, and two of these miRNAs (miR-146b-5p and miR-150) in the plasma of HIV/AIDS patients, including those on ART and those who developed ART resistance, to evaluate if these are biomarkers of disease progression and therapy.

**Results:**

We quantified miRNA levels by quantitative reverse transcription polymerase chain reaction (qRT-PCR) using RNA isolated from PBMCs and plasma of healthy persons or HIV-infected patients who were (1) asymptomatic; (2) symptomatic and ART naïve; (3) on ART; and (4) failing ART. Our results show miR-150 (p<0.01) and to a lesser extent miR-146b-5p (p<0.05) levels in PBMCs to reliably distinguish between ART-naïve AIDS patients, those on ART, and those developing drug resistance and failing ART. The plasma levels of these two miRNAs also varied significantly between patients in these groups and between patients and healthy controls (p values <0.05).

**Conclusions:**

We report for the first time that PBMC and plasma levels of miR-150 and miR-146b-5p are predictive of HIV/AIDS disease progression and therapy.

## Introduction

The pathogenesis of HIV/AIDS involves dynamic host-virus interactions, leading to a state of immune activation [1]. The median interval between HIV infection and the development of AIDS is 5–10 years in adults. Though not fully understood, variations in viral strains, host immune responses, microbial contact and environmental cofactors may contribute to this broad window. To assess disease progression and the efficacy of ART in patients with HIV-1 infection, three classes of surrogate markers have been used. These include HIV viral load, CD4+ T-cell numbers and plasma concentrations of soluble markers of immune activation, including Neopterin, Tumor Necrosis Factor alpha (TNFα), interleukins, beta 2-microglobulin, soluble CD8, etc. [2]. The best predictor for onset of AIDS is the percentage or absolute numbers of circulating CD4+ T cells in peripheral blood [3]. But, while T cell counts and viral loads are important predictors of disease progression, this has been questioned in patients on highly active anti-retroviral therapy (HAART) [4]. The CD4+ T-cell counts of HIV patients on HAART do not reliably identify individuals with virological failure [5]. A recent review of all the current biomarkers for HIV disease progression concluded that their clinical utility remained debatable [3]. Therefore, it is important to discover newer classes of biomarkers of early detection, disease progression and therapy.

Micro ribonucleic acids (miRNAs) are an abundant class of small RNAs of 18–25 nucleotides that post-transcriptionally regulate over 30% of the protein coding genes in humans [6]. In the most recent release, 2578 mature human miRNA sequences have been identified (Sanger miRBase release 20; http://www.mirbase.org/). During virus infection and its replication, host and viral RNAs and miRNAs interact in various ways, mutually regulating their levels and translational competence. Several reports on the differential expression of host and viral miRNAs and their roles in HIV infection were published recently [7]–[12]. While several small RNAs complementary to the HIV *env*, *nef* and/or LTR sequences directly inhibit viral replication *in vitro* [12] other miRNAs target critical host factors. For example, miR-17-5p and miR-20 inhibit HIV by reducing expression of the PCAF histone acetyltransferase [13]. Increased expression of miR-28, miR-150, miR-223 and miR-382 was recently credited for the inhibition of HIV-1 replication in monocytes [9]. A profiling study identified 62 differentially regulated miRNAs from the peripheral blood mononuclear cells (PBMCs) of HIV/AIDS patients with different CD4 counts and viral loads [10]. Of these, 59 miRNAs were down regulated and 3 were upregulated. Among the down regulated miRNAs, miR-16, miR-146b-5p, miR-150, miR-191 and miR-223 are abundantly expressed in B and T lymphocytes, and their levels correlated broadly with disease status [10]. Since imperfect complementarity allows a single miRNA to potentially target multiple mRNAs [14] and cellular mRNAs involved in the differentiation of hematopoietic cells and the regulation of immune cell function are major targets of miRNA-mediated regulation [15], we sought to assess the status of these five miRNAs during the progression of HIV infection and ART.

MicroRNAs are far more stable than mRNAs and have more plasticity in their cellular effects [14]. These have also been identified in the plasma and sera of healthy individuals and those with pathologic conditions, opening up the possibility of exploring miRNAs as disease biomarkers [16]. The evaluation of tissue-specific miRNAs in plasma has shown good promise as biomarkers in leukemia [17], [18], liver injury [19], viral hepatitis [20], [21] and cardiac disease [22]. With information from earlier studies on miRNA expression in HIV/AIDS [7]–[11] and other diseases [18]–[22], we hypothesized that differential regulation of miR-16, miR-146b-5p, miR-150, miR-191 and miR-223 [10] in PBMCs and plasma may be predictive of the status of HIV disease progression and response to therapy. In this pilot study, we have quantified the levels of these five miRNAs in PBMCs and that of miR-146b-5p and miR-150 also in the plasma of patients at different stages of HIV infection, on ART and those who showed ART resistance. To our knowledge, this is the first report of simultaneous miRNA measurements in PBMCs and plasma from HIV/AIDS patients on ART and those displaying resistance to ART. Our results show PBMC and circulating plasma miR-150 and to a lesser extent miR-146b-5p to be novel candidate biomarkers of HIV infection and disease.

## Results

### Study subjects

A total of 37 HIV-infected subjects (28 male, 9 female; average age 33 yr) at different stages of disease and treatment were included in this study. These were divided into Groups 1 to 4, as detailed in Methods. The distribution across groups and their median CD4 counts (number/ µl) and viral loads (copies/ml), was as follows: Group 1 (n = 8), CD4 = 497 [range 331–632], VL = 7716 [range 400–155719]; Group 2 (n = 10), CD4 = 152 [range 14–221], VL = 42272 [range 4728–167520]; Group 3 (n = 8), CD4 = 430 [range 282–702], VL = 11863 [range 453–64689]; and Group 4 (n = 11), CD4 = 126 [range 27–190]; VL = 24582 [range 7891–88538]. There were 9 healthy controls (average age 29 yr). All the patients on therapy received first line of ART, i.e. either Zidovudine (ZDV) + Lamivudine (3TC) + Nevirapine (NVP)/Efavirenz (EFV), or Stavudine (d4T) + 3TC + NVP/EFV. This was based on the ART recommendations of the National AIDS Control Organization (NACO), Government of India, prevailing at that time.

### Differential miRNA expression in PBMCs

**Figure 1:**
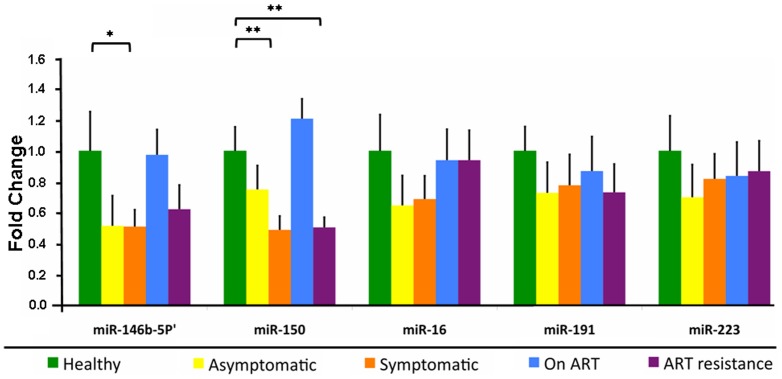
Differential expression of the indicated miRNAs in the PBMCs of healthy controls and HIV/AIDS patients at different stages of disease. The values in the bar graphs are given as mean ± SEM, and asterisks denote statistically significant differences (** p<0.01, * p<0.05) for the indicated groups (Student's T test).

Five miRNAs (miR-16, miR-146b-5p, miR-150, miR-191 and miR-223) were quantified in the PBMCs of HIV/AIDS patients at different stages of disease using TaqMan miRNA assays and compared to healthy controls. The miRNA expression levels were normalized using the stably expressed small nucleolar RNA44 (RNU44), and are shown as fold change ± standard error mean (±SEM) (Fig. 1). Though the five miRNAs were down regulated to various extents in HIV-infected persons when compared to healthy controls, only three of these reached statistical significance (Fig. 1). Though no significant change was observed in asymptomatic persons (Group 1) compared to healthy controls, miR-146b-5p and miR-150 were down regulated significantly to 0.51±0.08 fold (p<0.05) and 0.48±0.10 fold (p<0.01), respectively, in ART-naïve AIDS patients (Group 2). Following at least 6 months on ART (Group 3), the expression levels of both these miRNAs attained levels similar to healthy controls (Fig. 1). However, in patients who became resistant to first-line ART (Group 4), the expression levels of miR-146b-5p and miR-150 again fell to 0.62±0.15 fold (p>0.05) and 0.50±0.06 fold (p<0.01) respectively, compared to healthy controls. There were no significant differences in the mean cycle threshold (Ct) values of the endogenous control RNU44 (ANOVA; p-value 0.52).

### Relative expression of plasma miR-150 and miR-146b-5p

**Figure 2:**
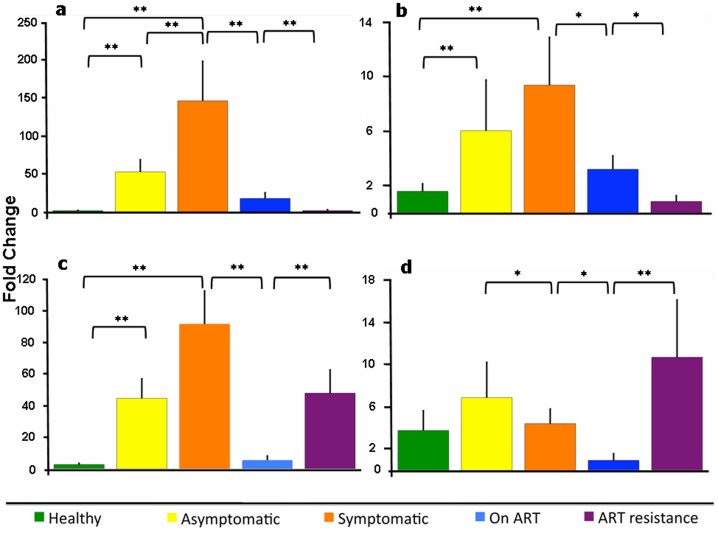
Relative plasma levels of miRNAs. The expression levels (mean ± SEM) of plasma miR-150 (a, b) and miR-146b-5p (c, d) normalized to miR-16 (a, c) and cel-miR-39 (b, d) in different groups of HIV patients and healthy controls are shown. The asterisks denote statistically significant differences (** p<0.01, * p<0.05) for the indicated groups (Student's T test).

Since miR-150 and miR-146b-5p are also reported as circulating miRNAs in plasma, we evaluated their plasma levels in the four groups of patients and in healthy controls. The levels of these miRNAs were normalized using the expression levels of miR-16, a widely used endogenous reference for the measurement of plasma miRNAs; the samples were also spiked with synthetic cel-miR-39 as an external control. Though the magnitudes of expression level changes were different, the same trend was observed regardless of whether miR-16 or cel-miR-39 was used as the normalizer (Fig. 2). The plasma levels of miR-150 (Fig. 2a,b) and miR-146b-5p (Fig. 2c,d) were significantly upregulated in the asymptomatic and symptomatic groups compared to healthy controls; in patients on ART these were similar to the levels in healthy controls. While miR-146-5p levels were significantly increased in the ART resistance group compared to patients on ART, we observed a further reduction in miR-150 levels in the ART resistance group.

### Absolute quantitation of miR-150 and miR-146b-5p in plasma

**Figure 3:**
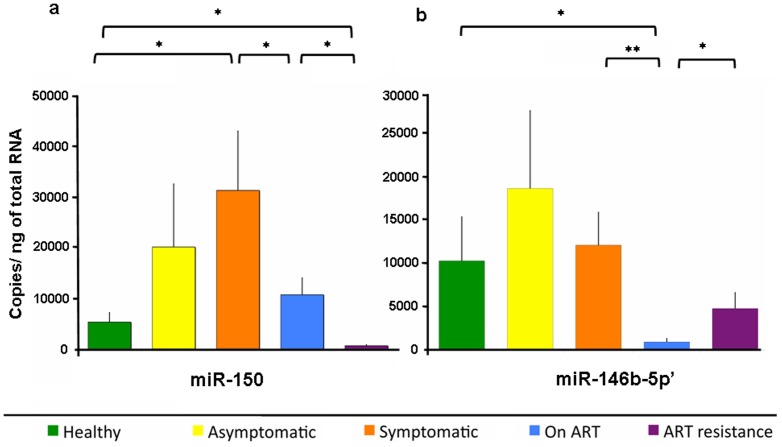
Absolute quantification of miRNAs in plasma. The levels of (a) miR-150 and (b) miR-146b-5p in different groups of HIV/AIDS patients and healthy controls by qRT-PCR are shown. The graph indicates the number of copies/ng of total plasma RNA. The error bar of each group represents mean ± SEM and asterisks denote statistically significant differences (** p<0.01, * p<0.05) for the indicated groups (Student's T test).

We then quantified the absolute amounts of these two miRNAs in the plasma from various groups of HIV/AIDS patients and healthy controls [27], [28]. The absolute amount of each miRNA was calculated with respect to standard curves based on serial dilution (10^9^ to 10^3^ copies) of spiked synthetic miR-150 or miR-146b-5p and analyzed using the same Taqman microRNA assay. The Ct values for each sample reaction were converted to absolute copy number based on these standard curves. All reactions were carried out in duplicate and the absolute quantities are presented as Mean ± SEM copies/ng total RNA (Fig. 3). Compared to healthy controls with 5307±1846 copies, the plasma levels of miR-150 were higher in asymptomatic (20138±12418), symptomatic (31310±11696) and ART (10705±3300) groups of patients; however, this reached significance only in the symptomatic group (p<0.01). Significantly lower levels of miR-150 were observed in the ART resistance (633±192) group compared to either healthy controls or patients on ART (p<0.05). The miR-150 levels were also significantly lower in the ART group when compared with the symptomatic group (p<0.05) (Fig. 3a). Compared to healthy controls with 10297±5069 copies, the plasma levels of miR-146b-5p were elevated in asymptomatic (18649±8982) and symptomatic (12106±3776) patients, but the changes were not significant. Patients on ART showed significantly lower plasma levels of miR-146b-5p (925±382) when compared to healthy controls and symptomatic patients. The patients with ART resistance had significantly higher levels of plasma miR-146b-5p (4781±1831) than patients on ART (p<0.05) (Fig. 3b).

### Correlation between PBMC and plasma miRNAs to CD4 cell counts and viral loads

**Figure 4:**
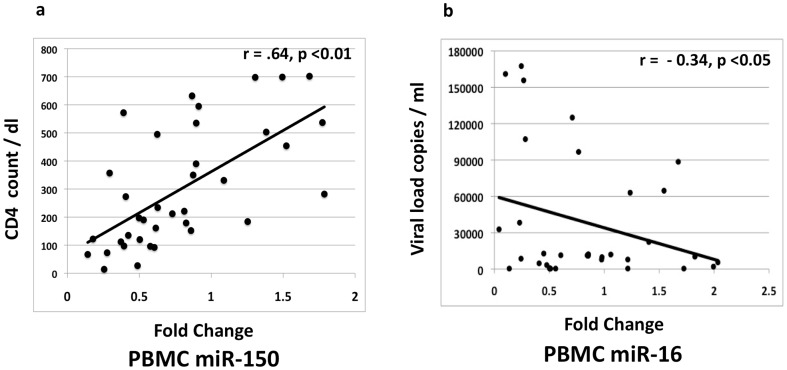
Correlation of miRNA levels with CD4 counts and viral load. The correlations are shown for (a) miR-150 in PBMCs with absolute CD4+ T-cell counts and (b) miR-146b-5p in PBMCs with HIV viral loads. A positive significant correlation was noted between miR-150 and CD4 cell count (r = 0.64; p<0.01) and an inverse correlation between miR-146b-5p and HIV viral loads (r = −0.34; p<0.05).

To determine whether any of the miRNA measurements could be utilized for monitoring HIV/AIDS patients with or without ART, we determined the correlation between miRNA levels and the existing surrogate markers - CD4+ T-cell counts or viral loads. Only 2^ΔΔCt^ value (or fold-change relative to healthy controls) for miR-150 in PBMCs positively correlated with CD4 cell counts across 37 samples belonging to different disease stages, with a Pearson correlation of 0.64 and p<0.01 (Fig. 4a). Thus, the expression levels of miR-150 increase with increasing CD4 counts. In the same manner, we found the relative expression of miR-146b-5p (Pearson correlation  = −0.36; p<0.05) (not shown) and miR-16 (Pearson correlation  = −0.34, p<0.05) (Fig. 4b) to correlate negatively with viral loads. The plasma levels (relative or absolute) of miR-150 and miR-146b-5p showed no significant correlation with CD4+ T-cell counts or viral loads. The miRNA correlations that were observed in PBMCs with CD4+ T-cell counts and viral loads were lost in plasma due to a further reduction in miRNA expression in the ART resistance group.

### Diagnostic accuracy of miR-150 and miR-146b-5p in PBMC and plasma as biomarkers

**Figure 5:**
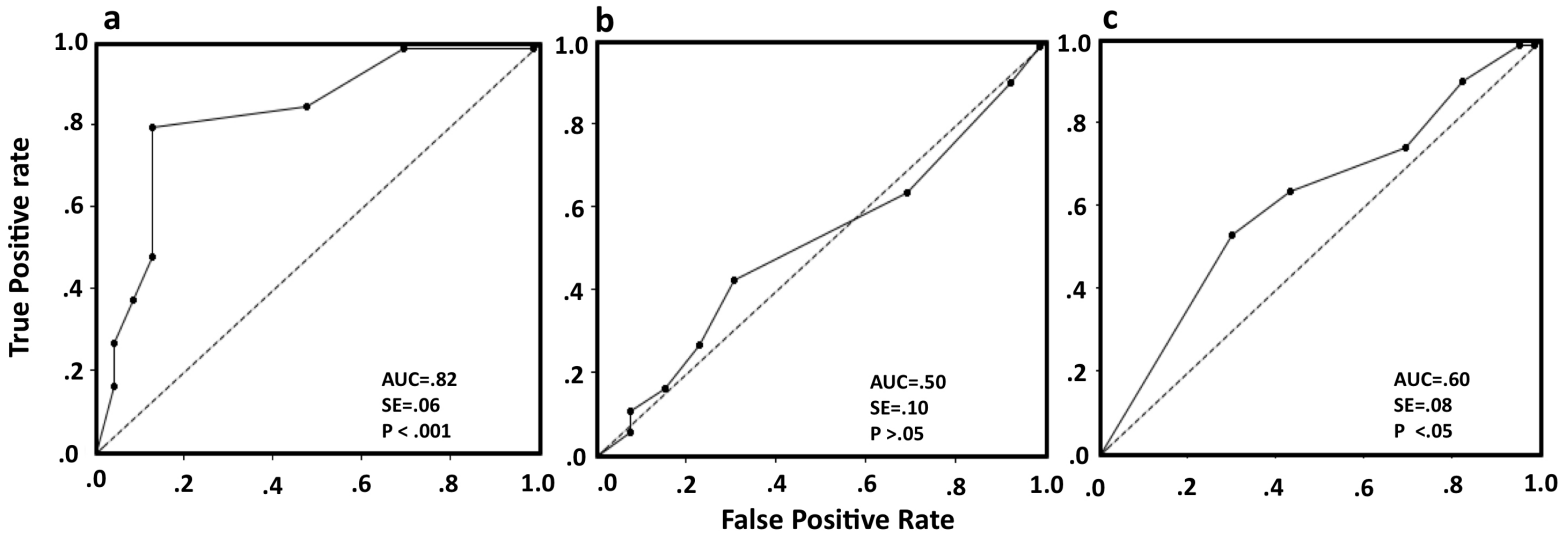
PBMC miRNAs as classifiers for different sets of patients. Receiver operating characteristic (ROC) curve analysis is shown for the expression ratio of (a) miR-150/RNU44 as classifier of CD4+ T-cell counts, (b) miR-146b-5p/RNU44 as classifier of CD4+ T-cell counts, and (c) miR-16/RNU44 as a classifier of viral load. Comparisons are made between Set-A (healthy, HIV+ asymptomatic and patients on ART) versus Set-B (HIV+ symptomatic and ART resistance patients), as described in the text.

We were interested in knowing whether the measured miRNA levels, especially for miR-150 and miR-146b-5p in PBMCs and plasma can differentiate between different stages of HIV disease. The expression value of each miRNA in each sample was calculated by dividing the Ct value of the target miRNA by the Ct value of the endogenous controls – RNU44 for PBMCs and miR-16 for plasma [29]. The study subjects were initially segregated into two sets. Set A included healthy controls, HIV-infected asymptomatic subjects (Group 1) and HIV-infected persons on ART (Group 3); Set B included HIV-infected symptomatic ART-naïve subjects (Group 2) and those failing first-line ART (Group 4). The diagnostic accuracy for predicting subjects belonging to Set A or Set B was evaluated by Receiver Operating Characteristic (ROC) curve analysis. The results showed the area under curve (AUC) value for PBMC miR-150 to be 0.82 (SE = 0.06, p<0.001) (Fig. 5a). In a similar analysis, PBMC miR-146b-5p showed an AUC value of 0.50 (SE = 0.10, p>0.05) (Fig. 5b). Since PBMC miR-16 levels showed significant correlation with viral loads, we also carried out ROC analysis for its ability to differentiate between Set A and B. This gave an AUC value of 0.60 (SE = 0.08, p<0.05) (Fig. 5c). The diagnostic accuracies of other individual miRNAs in PBMC as well as the combination of all five miRNAs were found to be poor (data not shown). The ROC analysis for copy numbers of plasma miR-150 and miR-146b-5p in these two sets showed AUC values of 0.50 and 0.60, respectively (p>0.05).

**Figure 6:**
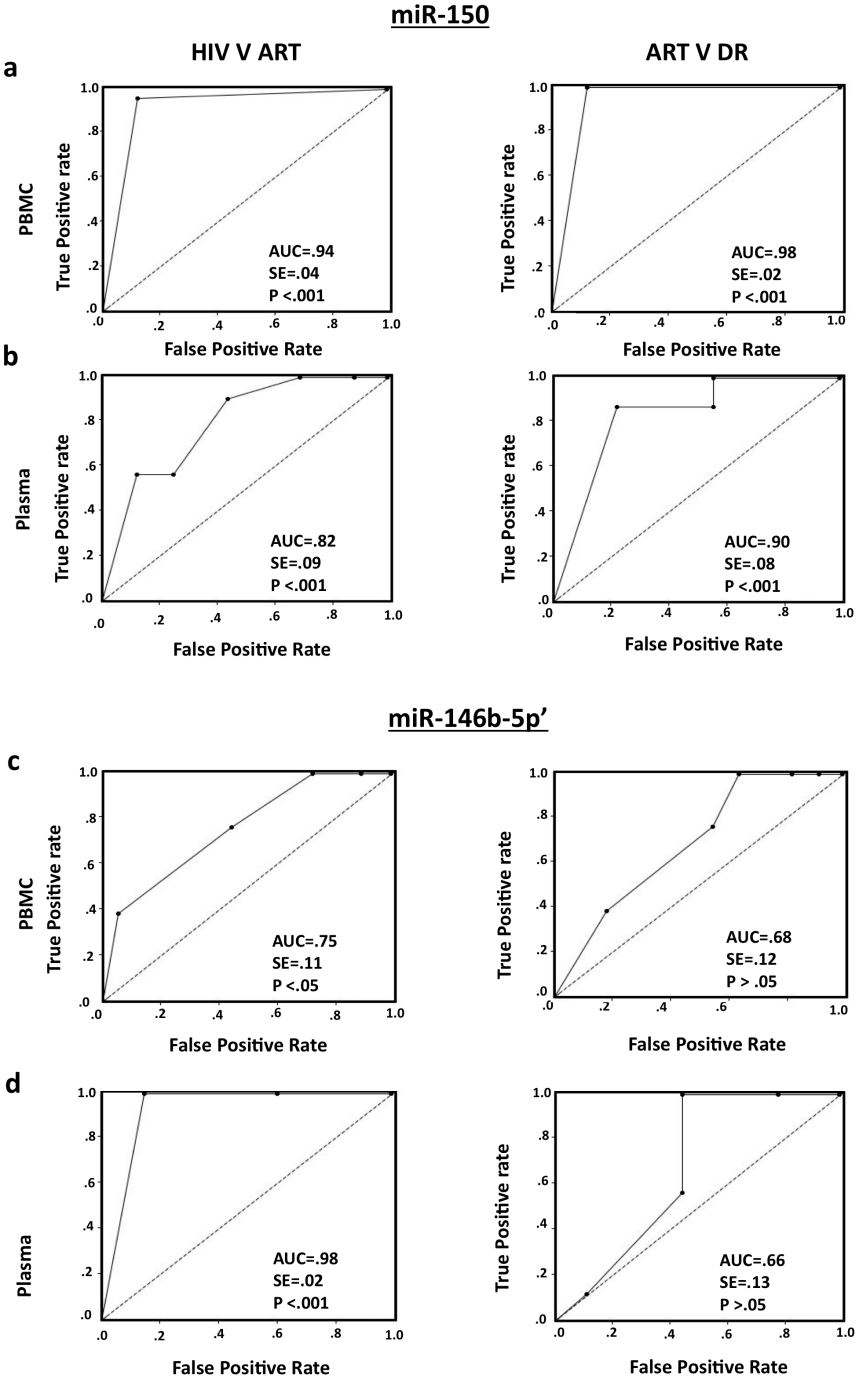
miRNAs as classifiers for patients without and with ART. Receiver operating characteristic (ROC) curve analysis of expression ratio of (a) PBMC miR-150/RNU-44, (b) plasma miR-150/miR-16, (c) PBMC miR-146b-5p/RNU-44 and (d) plasma miR-146b-5p/miR-16, as classifier of Set-C (HIV+; asymptomatic and symptomatic) versus Set-D (patients on ART) shown on left panels; and Set-D versus Set-E (ART resistance patients) shown on right panels. Insets of plots show area under curve (AUC), standard error (SE) and significance (p value).

To further test whether these miRNAs could differentiate HIV-infected asymptomatic and symptomatic ART-naïve subjects (Set C) from patients on ART (Set D), another ROC analysis was carried out. This showed the AUC values for PBMCs and plasma miR-150 to be 0.94 (SE = 0.04, p<0.001) and 0.82 (SE = 0.09, p<0.001), respectively (Fig. 6a,b; left panels). When we compared the patients on ART (Set D) with those failing first-line ART (Set E), the AUC values of PBMC and plasma miR-150 were 0.98 (SE = 0.02, p<0.001) and 0.90 (SE = 0.08, p<0.001) (Fig. 6a,b; right panels). The PBMC and plasma levels of miR-146b-5p showed high enough accuracy to differentiate between Set C and Set D with AUC values of 0.75 (SE = 0.11, p<0.05) and 0.98 (SE = 0.02, p<0.001), respectively; however, these did not accurately differentiate between patients in Set D and Set E (Fig. 6c,d). Together these results show PBMC and plasma miR-150 levels, and to a lesser degree miR-146b-5p levels to be good predictors of HIV infection and disease in pre- and post-ART patients.

## Discussion

We assayed five miRNAs in HIV/AIDS patients, which are expressed mainly in B and T lymphocytes, the major constituents of PBMCs, and were previously shown to correlate with CD4+ T-cell counts and viral loads in HIV infected persons [9], [10], [12]. Later, we also checked the plasma levels of two of these miRNAs (miR-150 and miR-146b-5p) that were differentially expressed in patients' PBMCs. Analysis of only selected miRNAs was a proof-of-principle study to assess their utility as alternative biomarkers, and to develop correlations between miRNA levels and disease status. The results presented in this report show miR-150 to potentially be a new biomarker for disease progression, therapy and resistance to therapy.

We found that miR-150 levels decreased in the PBMCs of HIV/AIDS patients; these were restored with ART but were further reduced in patients who developed drug resistance. On the other hand, miR-150 levels increased in patients' plasma and were reduced following ART and drug resistance. We also show that HIV positive individuals can be classified on the basis of the absolute quantities of miR-150 and miR-146b-5p, either in PBMCs or in plasma. Further, the ART status of patients can be determined from the levels of these miRNAs, especially that of miR-150 in PBMCs and plasma. The diagnostic accuracy was determined from a ROC analysis. It is generally accepted that AUC values of 0.70-0.90 represent medium accuracy and 0.90-1.00 signify high accuracy [30]. With AUC values of 0.94 and 0.82 respectively, miR-150 levels in PBMC and plasma appear to determine HIV disease progression with good precision. The PBMC and plasma levels of miR-146b-5p also provide decent correlation, except between patients on ART and those failing therapy.

Down regulation of miR-150 during HIV infection was reported earlier. Among elite controllers and viremic HIV patients, miR-150 levels in PBMCs showed positive correlation with CD4+ T-cell counts [7]. Swaminathan et al. reported lower levels of miR-150 in CD4+ T cells of chronic HIV patients compared to healthy controls [8]. It has been suggested that suppression of miR-150, which is usually expressed at high levels in monocytes, might facilitate HIV-1 infection [9]. It is a key regulator of immune cell differentiation and activation and is expressed in mature, resting B and T cells, but not in their progenitors. Ectopic expression of miR-150 in hematopoietic stem cell progenitors decreased the numbers of mature B cells by blocking the pro-B to pre-B cell transition [31]. MiR-150 controls c-Myb expression and affects lymphocyte development, with knockout mice showing increased naïve B cell expansion and antibody production [32]. Interestingly, during HIV infection, hyperactivated naive B cells are a major source of abnormal IgG production leading to hypergammaglobulinemia [33].

A recent report provides important insights into miRNA and mRNA deregulation during HIV infection, and showed the cellular transcriptome to be significantly modulated by HIV-1 through miRNAs [34]. However, unlike our and other studies, these authors did not observe changes in miR-150 or miR-14b-5p between uninfected healthy controls, infected persons with low viral load (<40 copies/ml) or high viral load (>50,000 copies/ml). These differences might be due to classification of patients based on the extremes of viral load [34] and not CD4 counts as in other studies.

Since our aim was to discover new miRNA biomarkers for HIV infection, we preferred to study this in PBMCs, which is an accessible cellular component, relates to immune responses and includes cells that are major targets of HIV [7]. Although the frequencies of different cell populations in PBMCs vary across individuals, it generally includes CD4+ T lymphocytes (25–60%), CD8+ T lymphocytes (5–30%), monocytes (10–30%), B cells (5–20%), NK cells (5–20%) and dendritic cells (1–2%). As miR-150 is present in CD4+ T lymphocytes [35], CD8+ T lymphocytes [36], monocytes [9] and B cells [37], we assume that the miR-150 that we measured mostly came from these cells, which constitute around 90% of PBMCs. Although the numbers of cells in PBMCs infected with HIV-1 varies from 0.1 to 13.5% [38], pronounced down regulation of miR-150 in this compartment reinforces the earlier speculation of bystander effects resulting from systemic changes in cellular activation, cytokine levels, etc. following HIV infection [11]. Elevated circulating lipopolysaccharide (LPS), which is correlated with depletion of CD4 cells during chronic HIV infection and AIDS [39], may also reduce miR-150 levels in leukocytes [40].

It was shown earlier that miRNAs are stably expressed in animal serum/plasma and that their unique expression patterns may serve as “fingerprints” for a number of diseases, especially various cancers [41]. Therefore, we examined whether the levels of miR-150 and miR-146b-5p showed similar trends in plasma as in PBMCs. Contrary to PBMCs, we found increased levels of these two miRNAs in the plasma of symptomatic patients, with the levels reducing on ART and ART resistance. Though most studies found the same trend of alteration between circulating miRNAs and tissue miRNAs [42], [43], the opposite is also true [44]. With the initiation of ART, miR-150 levels in PBMCs returned to normal, and on ART resistance these again reduced significantly. There could be multiple reasons for this, including epigenetic changes during prolonged ART administration. We have also observed gene expression patterns and cytokine levels in ART resistant patients to sometimes be opposite of what is expected (S. Munshi, et al; unpublished data).

With reduced levels of these miRNAs in the PBMCs of HIV/AIDS patients, we expected to see that in plasma as well. But since circulating miRNAs are derived not only from circulating peripheral blood cells but also from other tissues affected by the infection/disease [41], we assume that the increase in circulating miR-150 is due to cellular sources other than PBMCs. Circulating miRNAs are either released due to cytolysis or tissue injury, in apoptotic bodies or actively secreted from cells in small membranous shedding vesicles, called exosomes and microvesicles, or as RNA-protein complexes [45]. While miR-150 and other miRNAs are synthesized in different cells and tissue, it would be interesting to define the sources of these circulating miRNAs in HIV/AIDS. The high levels of Ceramide in HIV-infected cells [46], which promote exosome secretion [47], or ongoing apoptosis in bystander cells could be possible sources of increased circulating miR-150 and miR-146b-5p in HIV patients. Ceramide is a bioactive sphingolipid that is increased when cultured neurons are exposed to HIV gp120 and Tat proteins, as well as in the brain tissue and cerebrospinal fluid of patients with HIV-associated dementia [48]. Proinflammatory cytokines like tumor necrosis factor-α (TNF- α) and interleukin-1β, which are produced in high quantities during HIV infection, can also induce the generation of Ceramide and apoptosis in brain cells [49], and play a role in increased secretion of miRNA containing exosomes or apoptotic bodies.

## Methods

### Study subjects

Whole blood was collected from HIV/AIDS patients recruited from the National AIDS Control Organization ART Clinics at Dr. Ram Manohar Lohia Hospital and Maulana Azad Medical College Hospital in New Delhi, India. Ethics committees at the participating institutions and the National AIDS Control Organization (NACO), New Delhi, India, approved the study. Written informed consent was obtained from each participant before obtaining the samples. All subjects were HIV seropositive; those on anti-tubercular therapy were excluded from the study. They were divided into four groups based on their CD4 counts and ART status, as follows: Group 1, CD4 >350/ µl and ART naïve (asymptomatic); Group 2, CD4 <200/ µl and ART naïve (symptomatic); Group 3, CD4 >250/ µl and receiving ART for at least 6 months (on ART); and Group 4, CD4 <200/ µl and resistant to first-line ART (ART resistance). Patients in different groups were selected to ensure no overlap in the CD4 counts to keep the groups distinct. The clinical, immunological and ART data on subjects was collected from the participating hospitals.

### RNA preparation from PBMC

Peripheral blood mononuclear cells were isolated using Ficoll-Hypaque from 5 ml of blood, which was collected in K+EDTA-coated vacutainers (Becton Dickinson, Franklin Lakes, NJ, USA). The isolated PBMCs were stored at −80°C till further use. Total RNA was extracted from PBMCs with the RNeasy Mini Kit (Qiagen, Germany) according to the manufacturer's instructions. The RNA concentration was estimated on a NanoDrop 1000 (Thermo Scientific, Wilmington, DE, USA); representative samples were also randomly checked for RNA integrity and concentration by capillary electrophoresis on an Agilent 2100 Bioanalyzer (Agilent Technologies, Inc, Santa Clara, CA).

### RNA preparation from plasma

Total RNA was isolated from plasma using the miRNeasy kit (Qiagen, Germany) with few minor modifications. In brief, 600 µl of QIAzol reagent was added to 100 µl of plasma sample. The sample in the tube was mixed, followed by addition of 3.5 µl (1.6x10^8^ copies/ µl) of *C. elegans* miR-39 (Qiagen, Germany), and 140 µl of chloroform. After vigorous mixing for 15 seconds, the plasma sample was centrifuged at 12,000xg for 15 min and the upper aqueous phase was transferred to a new tube. To this 1.5 volumes of ethanol were added and the sample was applied directly to the column. The immobilized RNA was eluted in 50 µl of RNAse-free water and was quantified using a NanoDrop 1000 spectrometer (Thermo Scientific, Wimington, DE, USA). The efficiency of small RNA isolation was monitored by the amount of spiked-in *C. elegans* miR-39 recovered and was used as internal control for normalizing the expression of miR-146b-5p and miR-150.

### miRNA assay

The miRNAs were assayed individually in each sample using TaqMan MicroRNA Assays (Applied Biosystems, Foster City, CA, USA) according to the manufacturer's protocol. For synthesis of each miRNA-specific cDNA, 10 ng of total RNA was reverse transcribed using TaqMan miRNA reverse transcription kit (Applied Biosystems, Foster City, CA, USA) in a 15 µl reaction volume containing 1X RT buffer, 0.15 µl of 100 mM dNTPs (with dTTP), 0.19 µl of RNase inhibitor (20 units/ml), 1 µl of MultiScribeTM Reverse Transcriptase (50 units/ml) and 3 µl of each of the miRNA specific stem-loop primers. The primers used were: hsa-miR-16, 000391; hsa-miR-146-5b, 001097; hsa-miR-150, 000473; hsa-miR-191, 2299; hsa-miR-223, 000526; and cel-miR-39, 000200 (Applied Biosystems, Foster City, CA, USA). The mixture was incubated at 16°C for 30 min, 42°C for 30 min and 85°C for 5 min. Quantitative real-time PCR was then carried out on the StepOne Plus cycler (Applied Biosystems). Briefly, each 20 µl reaction consisted of 2.5 µl of the reverse transcription product, 10 µl TaqMan 2X Universal PCR Master Mix No AmpErase UNG, 1 µl TaqMan MicroRNA Assay (20X) containing the TaqMan primer-probe mixture. Reactions were initiated with a 10 min incubation at 95°C followed by 40 cycles of 95°C for 15 sec and 60°C for 60 sec. Small nucleolar RNA 44 (RNU44) was used as an endogenous control to normalize miRNA expression in PBMC as used by others [17], [23]. In the plasma cDNA we also measured the levels of endogenous miR-16 and spiked-in synthetic cel-miR-39 and these were used as internal controls to normalize the expression of miR-150 and miR-146b-5p [18]. To determine the absolute copy number of miR-150 and miR-146b-5p, standard curves was prepared for each miRNA by serial dilution of synthetic miR-150 or miR-146b-5p (Sigma Aldrich, Bangalore, India), taking 10^3^, 10^5^, 10^7^ and 10^9^ copies for each miRNA specific reverse transcription reaction in separate tubes followed by its real-time PCR reaction using respective TaqMan MicroRNA assays. All the experiments were performed in duplicates for each sample and reverse transcriptase-negative controls were included in each batch of reactions.

### Viral load measurement

HIV-1 plasma viral loads were quantitated by an in-house reverse-transcriptase TaqMan real-time PCR assay using specific primers and probes from a conserved region of the *gag* gene [24]. Briefly, viral RNA was isolated from 100 µl of plasma by QIAamp Viral RNA Mini Kit (Qiagen, Germany) and subjected to reverse transcription using SuperScript III reverse transcriptase as per manufacturers protocol. The real-time PCR was performed on a StepOne Plus cycler using HIV-1 primers and an internal TaqMan probe (Applied Biosystems). A plasma sample that contained 150,000 copies/ml (NIH AIDS Reagent Bank) was similarly treated and used to obtain the standard curve.

### Statistical analysis

The relative expression levels of miRNAs were calculated using the comparative ΔΔCt method as described previously. The fold changes in miRNAs were calculated by the equation 2^−ΔΔCt^ [25], [26]. Expression data were presented as mean ± standard error of mean (SEM) with 2-tailed p values. Correlation analysis was performed using two-tailed Pearson correlation test. Sensitivity, specificity, and the area under the curve (AUC) for specific miRNAs were estimated using Receiver Operator Characteristic (ROC) analysis using “ROC Analysis” software (Watkins, M. W. 2002, State College, PA: Ed & Psych Associates). Data were analyzed using Student's T or ANOVA tests, and p-values of 0.05 or lower were considered to be significant.

## Conclusions

With the aim of identifying new biomarkers of HIV infection and disease, we evaluated the levels of select miRNAs in PBMCs and plasma of HIV/AIDS patients. An ideal biomarker should not only indicate disease progression, response to therapy and failure of therapy, but should also have a role in the natural history of infection and disease. From that point, this pilot study showed expression levels of miR-150 in PBMCs and plasma could be a good indicator of the status of HIV disease.
